# Socio-demographic characteristics of Danish blood donors

**DOI:** 10.1371/journal.pone.0169112

**Published:** 2017-02-09

**Authors:** Kristoffer Sølvsten Burgdorf, Jacob Simonsen, Anna Sundby, Klaus Rostgaard, Ole Birger Pedersen, Erik Sørensen, Kaspar René Nielsen, Mie Topholm Bruun, Morten Frisch, Gustaf Edgren, Christian Erikstrup, Henrik Hjalgrim, Henrik Ullum

**Affiliations:** 1 Department of Clinical Immunology, Copenhagen University Hospital, Rigshospitalet, Copenhagen, Denmark; 2 Department of Epidemiology Research, Statens Serum Institut, Copenhagen, Denmark; 3 Department of Clinical Medicine, University of Aarhus, Aarhus, Denmark; 4 The Lundbeck Foundation Initiative for Integrative Psychiatric Research, iPSYCH, Copenhagen, Denmark; 5 Department of Clinical Immunology, Naestved Hospital, Naestved, Denmark; 6 Department of Clinical Immunology, Aalborg University Hospital, Aalborg, Denmark; 7 Department of Clinical Immunology, Odense University Hospital, Odense, Denmark; 8 Denmark and Department of Clinical Medicine, Center for Sexology Research, Aalborg University, Aalborg, Denmark; 9 Department of Medical Epidemiology and Biostatistics, Karolinska Institutet, Stockholm, Sweden; 10 Hematology Centre, Karolinska University Hospital, Stockholm, Sweden; 11 Department of Clinical Immunology, Aarhus University Hospital, Aarhus, Denmark; 12 Department of Hematology, Copenhagen University Hospital, Rigshospitalet, Copenhagen, Denmark; Goethe University Medical School, GERMANY

## Abstract

**Background:**

Blood transfusion is an essential component of a modern healthcare system. Because knowledge about blood donor demography may inform the design of strategies for donor recruitment and retention, we used nationwide registers to characterize the entire population of blood donors in Denmark in 2010.

**Methods:**

The study population comprised all Danes in the age range eligible for blood donation (N = 3,236,753) at the end of 2010. From the Scandinavian Donations and Transfusions (SCANDAT) register, we identified 174,523 persons who donated blood in Danish blood banks at least once in 2010. The association between sociodemographic characteristics and blood donor prevalence was examined using regression models.

**Results:**

The overall prevalence of blood donation was 5.4% among both women and men. The age-specific prevalence of blood donation peaked at 25 years of age (6.8%) for women and 30 years of age (5.7%) for men. Children of any age were associated with lower prevalence of blood donation among women, while the opposite was seen for men. Middle to high income groups, but not the highest income group, had fourfold higher donor prevalence than the lowest income group (6.7% compared to 1.7%). The prevalence of blood donation was considerably lower among men living with their parents (2.9%) or alone (3.9%) than among men cohabitating with a woman (6.2%).

**Summary:**

Social marginalization, as indicated by low income and being a male living without a woman, was associated with lower prevalence of blood donation. However, individuals with very high incomes and women with children were underrepresented in the Danish blood donor population.

## Introduction

Blood transfusions continue to play an important role in modern health care. To satisfy the need for a safe and efficient blood supply, it is essential to not only retain active donors but also to continuously recruit new donors to replace those who retire from donation.

Donor recruitment efficacy is optimized by targeting those segments of the population with the largest available resource and by focusing on those who are the most likely to respond positively. In this regard, it is reasonable to assume that potential donors would be similar to already active donors with respect to age, sex and sociodemographic characteristics. Therefore, donor recruitment efforts may benefit from detailed knowledge about demographic characteristics of both donors and non-donors, i.e., factors that are related to the probability of being a blood donor.

In Denmark 300,000 blood donations are collected annually from approximately 230,000 donors (4.1% of the Danish population aged 17–67). The recruitment of blood donors is organized by a nationwide organization “Danish Blood Donor Association”. The Danish healthcare system is tax financed, administrated in five health care regions by democratically elected assemblies. The Danish blood banks are integrated with the hospital system in each of the five administrative regions, and collect blood at 29 hospitals in addition to regional mobile donation units covering 180 different sites nationally (e.g. large companies and universities) [1]. The blood banks coordinate their work including donor recruitment through the Organization of Transfusion Centers in Denmark.

In recent years, there has been a growing literature describing sociodemographic characteristics of blood donors all over the world. Within the last decade, several countries have been in the process of changing the profile of blood donors from remunerated to non-remunerated [2; 3; 4; 5]. Large studies have been conducted to increase the knowledge of donor profiles to target inclusion strategies towards specific groups defined by e.g. age, gender, income, education and ethnicity.

Several nationwide studies have compared blood donors to the general population [6; 7; 8]. The many investigations of donor demographics have not revealed a clear picture of the typical donor. With respect to age of the donors, previous studies have found blood donor populations either to be younger [9; 4; 10; 8] or older compared to the general population [11; 12; 13; 14].

The same diversity concerns the donor gender composition. Several studies report men to have a higher donor prevalence than women [4; 10; 15], but the nationwide study from Great Britain reported that 55% of their donors where women [7]. Studies have also generally shown that those of higher socioeconomic status, whether measured by education or personal income, are more likely to be blood donors than individuals with lower status [10; 16; 12; 9; 17], although Carneiro-Proietti et al. reported a lower prevalence of donors with higher education [4]. Finally, although ethnic minority groups are growing in numbers in many countries [7] there is a clear picture that they are markedly underrepresented in the blood donor population [9; 18; 19].

The aim of this study was to provide an unbiased nationwide sociodemographic description of blood donors to allow blood banks to take this information into consideration when designing strategies for future blood donor retention and recruitment. We utilized existing national demographic registers to compare the entire population of blood donors in Denmark in 2010 with the entire non-donor Danish population.

## Materials and methods

In Denmark, all individuals are uniquely identifiable through 10-digit Civil Registration System (CRS) numbers which have been assigned to all residents since 1968. By means of the CRS numbers, the CRS continuously monitors the vital status of all individuals living in Denmark and records information about family relations and residence. The last all-encompassing collection of data from the Danish blood banks with subsequent data handling comparing with the entire Danish population was done in 2011.

Within the CRS, we identified all individuals potentially eligible for blood donation in 2010, i.e., those age 18–65 years and residing in Denmark as of December 31, 2010. For these individuals, we extracted information on sex; age; ethnicity (born in Denmark by at least one ethnically Danish parent, born in Denmark by non-Danish parents, born in a Western country other than Denmark, or born in a non-Western country); parental birth place; cohabitation status (living with parents, living alone, living with a person of opposite gender, living with a person of same gender, living in a multi-household which is a household with three or more unrelated adults); age of youngest child in the household (0, 1–2, 3–5, 6–8, 9–11 or 12+ years old, or no children); and level of urbanization (<25, 25–350, >350–1000, >1000–2000, >2000 persons per square kilometer).

Using CRS numbers as identifiers, we linked the population of potentially eligible blood donors in 2010 to nationwide registers maintained by Statistics Denmark [20]. Here, we were able to obtain individual information on education (primary and lower secondary education, high school, technical and vocational education and training, higher short/middle length education, higher long term education), and income for the year 2008. We decided to use the data for income and education before donation to make sure, that pregnancy and maternity/paternity leave did not affect data for income.The income variable was calculated as deciles relative to gender and birth year.

Finally, we took advantage of The Scandinavian Donations and Transfusions (SCANDAT) database to identify individuals who had donated blood in a Danish blood bank in 2010. In Denmark, computerized registration of blood donors was introduced locally in 1981 and gradually expanded to reach national coverage by 2003 [20]. All available information from such databases in Denmark has been amalgamated in the SCANDAT II database as previously described [21]. Because all donors are identified by their CRS numbers, we were able to distinguish between donors and non-donors by linking SCANDAT II with the established data set. Only whole blood donors with a successful blood donation in 2010 were included in the study.

Binary regression models with log-link were used to estimate relative risk (RR) for blood donation for demographic/sociodemographic variables. Maximum likelihood estimates of the RRs and 95% confidence intervals were calculated in mutually adjusted analyses, i.e. from one joint model. Data are presented with prevalence of blood donation with mutually adjusted relative risk and 95% confidence intervals e.g. 5.5%, 1.20(1.16–1.23), compared to the specified reference group e.g. 5.7%, reference group. The relative risk for each subgroup is compared to the reference group (RR = 1.00). If the relative risk for a subgroup is e.g. 1.20 the group has a 20% increased relative risk of donating blood. All analyses were adjusted for age. Because of the large nationwide dataset, 95% confidence intervals were very narrow and most of the p-values very low. We did not correct for multiple testing and only confidence intervals are shown.

To show the prevalence as a smooth function of age, we identified the number of donors and number of eligible donors at each age in days. The prevalence was then smoothed using the loess algorithm [22]. Statistical analyses were conducted using SAS 9.3 (SAS Institute Inc., Cary, NC, USA).

## Results

Overall, we identified 3,411,276 persons between 18 and 65 years of age who were living in Denmark on December 31, 2010. Of these, 174,523 (5.4% for both men and women) donated blood at least once in 2010.

### Age

The age-specific prevalence of blood donors differed between women and men. Among women, the prevalence and relative risk of blood donation was highest at 25 years of age (7.5%, 1.50 (1.45–1.55)) and decreased to 5.5%, 1.20 (1.16–1.23) at 30 years of age. The reference group for both men and women was 40 years of age.

**Fig 1:**
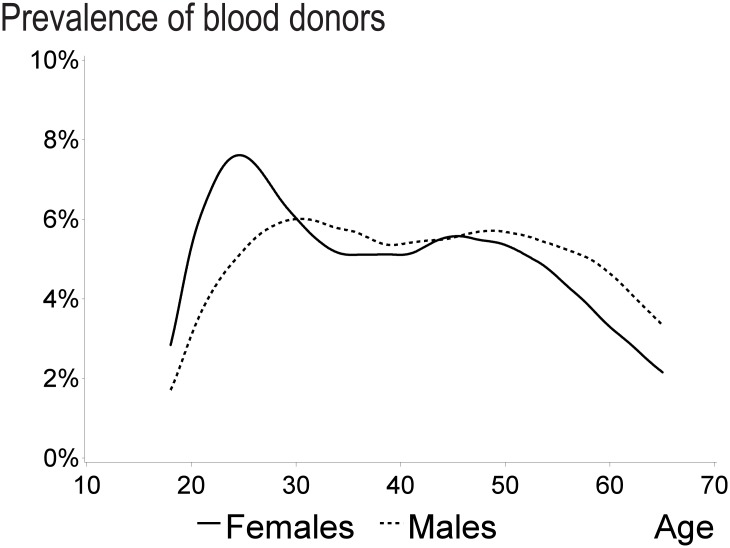
Prevalence of blood donors by sex and age.

The prevalence and relative risk was stable for women between the ages of 30 and 50 years and decreased gradually thereafter to 2.2%, 0.45(0.44–0.47) among 65-year-old women (Fig 1).

For men, donor prevalence plateaued at approximately 5.5% for all age groups between 25 and 55 years, and decreased thereafter. Compared to women, above age 55 years blood donor prevalence and relative risk among men were higher, e.g. at the maximum age for donation of 65 years (3.8%; 0.62(0.60–0.64)) (Fig 1).

### Children—age of youngest child

Overall, having children affected men’s and women’s donor prevalence and relative risk differently, and the age of the youngest child also seemed of importance. Having no children in the household was associated with the highest prevalence and relative risk of blood donation in women (6.2%, reference group), and with the lowest donor prevalence and relative risk in men (4.2%, reference group) (Table 1). Women have six months quarantine after pregnancy and cannot donate while breastfeeding, which plausibly explains the very low donor prevalence and relative risk among those whose youngest child was 0 years old (1.3%, 0.15(0.14–0.16)). Among women, the relative risk increased with increasing age of the youngest child (Table 1). For men, the prevalence and relative risk of blood donation were highest in the group of men with children above the age of 12 (5.6%, 1.24(1.21–1.28)), which also was the largest donor group. In contrast to women, the prevalence and relative risk of blood donation were slightly increased for men with children 0 years old (6.7%, 1.06(1.02–1.10)).

**Table 1:** Characteristics of Danish blood donors (174,523) vs. the general Danish population of non-donors (3,411,276). Included individuals between 18 and 65 years of age in Denmark in 2010. All analyses were adjusted for age.

	Women				Men			
Educational level	Non-donor	Donor	%	Adjusted RR (95% CI)	Non-donor	Donor	%	Adjusted RR (95% CI)
Primary and lower secondary education	458,217	18,686	3.9	0.60 (0.59–0.62)	486,212	16,812	3.3	0.48 (0.47–0.49)
High school	112,433	10,156	8.3	1.19 (1.16–1.23)	86,536	6,436	6.9	1.00 (0.97–1.03)
Vocational education	483,167	29,790	5.8	1.03 (1.01–1.05)	572,069	37,490	6.2	0.84 (0.82–0.85)
Higher education, short/middle length	340,627	21,040	5.8	Ref	235,925	18,160	7.1	Ref
Higher education, long	85,271	4,749	5.3	0.89 (0.86–0.92)	106,724	7,281	6.4	0.91 (0.89–0.94)
Unknown	128,078	2,202	1.7	0.41 (0.39–0.44)	141,494	1,721	1.2	0.32 (0.30–0.34)
***Cohabitation status***								
Living with parents	88,517	4,573	4.9	0.88 (0.85–0.92)	140,335	4,147	2.9	0.58 (0.56–0.60)
Single	414,912	21,224	4.9	0.85 (0.83–0.86)	408,733	16,708	3.9	0.69 (0.68–0.71)
Living with person of opposite gender	1,026,922	56,621	5.2	Ref	960,826	63,724	6.2	Ref
Living with person of same gender	25,810	2,102	7.5	1.12 (1.07–1.18)	42,304	1,389	3.2	0.59 (0.56–0.62)
Multi-household	51,632	2,103	3.9	0.82 (0.79–0.86)	76,762	1,932	2.5	0.62 (0.59–0.65)
***Age of youngest child***								
No children	485,182	32,148	6.2	Ref	655,161	28,844	4.2	Ref
0 years old	61,886	818	1.3	0.15 (0.14–0.16)	57,061	4,114	6.7	1.06 (1.02–1.10)
1–2 years old	107,669	6,196	5.4	0.69 (0.67–0.72)	104,308	6,790	6.1	0.98 (0.95–1.01)
3–5 years old	111,676	6,244	5.3	0.76 (0.74–0.79)	108,807	6,399	5.6	0.96 (0.93–0.99)
6–8 years old	91,146	5,051	5.3	0.82 (0.79–0.84)	88,694	5,349	5.7	1.05 (1.01–1.08)
9–11 years old	89,649	5,165	5.4	0.89 (0.86–0.92)	86,751	5,299	5.8	1.09 (1.06–1.13)
12+ years old	660,585	31,001	4.5	1.00 (0.97–1.02)	528,178	31,105	5.6	1.24 (1.21–1.28)
***Urbanization level***								
<25 person/km^2^	70,938	3,584	4.8	0.89 (0.86–0.93)	80,928	3,686	4.4	0.76 (0.74–0.79)
25–350 person/km^2^	229,112	11,336	4.7	0.87 (0.85–0.89)	248,966	12,130	4.7	0.79 (0.78–0.81)
350–1000 person/km^2^	281,821	14,785	5.0	0.92 (0.90–0.94)	277,853	16,035	5.5	0.90 (0.88–0.92)
1000–2000 person/km^2^	338,471	18,080	5.1	0.95 (0.94–0.97)	328,800	19,545	5.6	0.97 (0.95–0.99)
2000+ person/km^2^	658,770	37,635	5.0	Ref	656,309	35,184	5.1	Ref
Unknown	28,681	1,203	4.0	0.78 (0.74–0.83)	36,104	1,320	3.5	0.77 (0.73–0.82)
***Ethnicity***								
Born in Denmark with a Danish parent	1,380,148	83,303	5.7	Ref	1,403,748	84,818	5.7	Ref
Born in Denmark with no-Danish parents	19,299	456	2.3	0.39 (0.36–0.43)	26,451	601	2.2	0.52 (0.48–0.56)
Born in a non-Western country	146,197	1,032	0.7	0.17 (0.16–0.18)	134,166	871	0.7	0.17 (0.15–0.18)
Born in another Western country	57,140	1,692	2.9	0.71 (0.68–0.75)	58,991	1,444	2.4	0.59 (0.56–0.62)
Unknown	5,009	140	2.7	0.63 (0.54–0.75)	5,604	166	2.9	0.64 (0.55–0.74)

### Income

**Fig 2:**
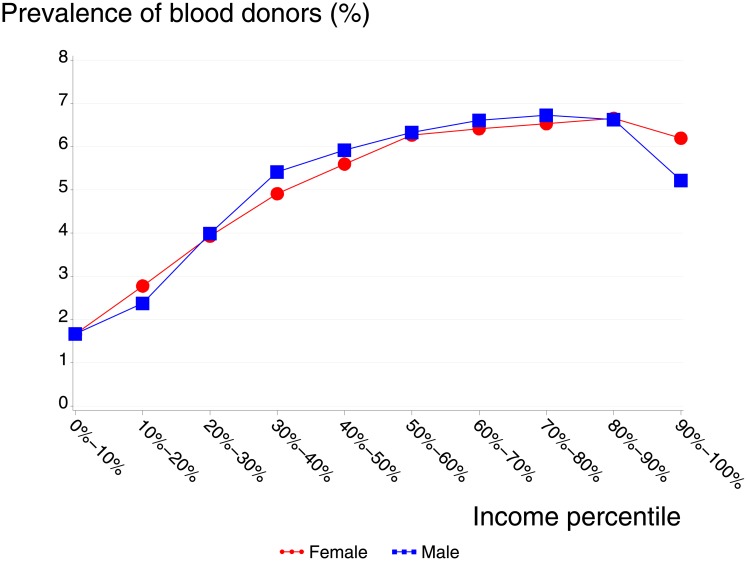
Prevalence of blood donors by income and age.

Fig 2 shows the prevalence of donors by deciles of personal income. The pattern was similar for women and men; the prevalence and relative risk of blood donors was lowest for the lower income groups, starting with 1.7%, 0.43(0.41–0.45) for the lowest income decile compared to income group five as reference group. Blood donor prevalence and relative risk increased for both women and men with increasing income, peaking at 6.5%, 1.17(1.13–1.20) in the 70–90% personal income deciles. There was a fourfold difference between those in the lowest income group compared with those in the middle to high income group. For both women and men, donor prevalence was lower among individuals in the highest 10% income group compared to the peak in the 70–90% personal income deciles. For women the relative risk was still above the average ((women 6.2%, 1.10(1.07–1.13), while the prevalence and relative risk for men in the 10% highest income group were lower (5.2%, 0.74(0.72–0.76)) compared to the overall donation prevalence of (5.4%) (Fig 2).

### Education

For both sexes donation prevalence and relative risk were lowest among persons with the lowest education level (women 3.9%, 0.60(0.59–0.62); men 3.3%, 0.48(0.47–0.49)) or an unknown level of education (women 1.7%, 0.41(0.39–0.44), men 1.2%, 0.32(0.30–0.34)) compared with the reference category of persons with short or middle length higher education (Table 1). Among women, the highest donor prevalence and relative risk were seen in those with a high school education (8.3%, 1.19(1.16–1.23)). Among men, the donor prevalence and relative risk were highest among those with a short/middle length of education (7.1%, reference group).

### Cohabitation

The prevalence of blood donors varied considerably by cohabitation status (Table 1). Among women, the highest prevalence and relative risk of donors (7.5%, 1.12(1.07–1.18)) were observed in the relatively small group of women, who lived with another woman. The prevalence and risk varied little between women living with a man (5.2%, reference group), women living with their parents (4.9%, 0.88(0.85–0.92)) and women living alone (4.9%, 0.85(0.83–0.86)).

Among men, the highest prevalence (6.2%, reference group) was observed among those who lived with a woman, with considerably lower prevalence and relative risk in all other cohabitation groups. The second highest prevalence and relative risk (3.9%, 0.69(0.68–0.71)) were observed among men, who lived alone, followed by men living with their parents (2.9%, 0.58(0.56–0.60)), men living with another man (3.2%, 0.59(0.56–0.62)); and men living in a multi-household (2.5%, 0.62(0.59–0.65)).

### Level of urbanization

The prevalence and relative risk of blood donors living in rural areas were lower than the prevalence of blood donors in the most urbanized areas both in men and women (Table 1). The differences of the relative risk between rural and urbanized areas were greatest for men, with the lowest prevalence and relative risk among men in rural areas (<25 person/km^2^ 4.4%, 0.76(0.74–0.79). For women, the tendency was the same but not as marked (women 4.8%, 0.89(0.86–0.93)) compared to the reference group 2000+ person/km^2^ (Table 1).

### Ethnicity

Danes with Danish parents had the highest prevalence and relative risk of blood donation (5.7%, reference group) in both women and men. The prevalence and relative risk were considerably lower for Danes with no Danish parents (2.3%, 0.39(0.36–0.43) and 2.2%, 0.52(0.48–0.56) for women and men, respectively). The prevalence and relative risk of blood donors from the large group of people born in Non-Western countries was extremely low (0.7%, 0.17(0.16–0.18)) and (0.7%, 0.17(0.15–0.18) for women and men, respectively) (Table 1).

## Discussion

We used the unique Danish registry resources to characterize the sociodemographic profile of the entire Danish blood donor population of 2010. Among our more salient observations, we note that blood donor prevalence and relative risk were higher among young women than among young men and that individuals with shorter education and lower income were underrepresented in the donor population. The same was true for men from the highest income groups, those of non-Danish descent, and men who did not live with a woman.

The observed decrease in donor prevalence after the age of 25 years most likely reflects childbearing and breastfeeding. Interestingly, however, parenthood seemed to have the opposite effect on men, in whom having young children was associated with an increased prevalence of donation. Both women and men donated at increasing prevalence with increasing age of children.

Data from the *Healthy People 2010* study showed that individuals between 18 to 24 years of age had a donation prevalence of 8%, while individuals between 25 and 64 years of age had a lower donation rate of 6% [9]. Our data shows the opposite, with donation rates of 4.9% for the 18 to 24 years age group and 5.2% for the 25 to 64 years age group. The difference reflect the fact, that the blood supply in Denmark is based mainly on repeat donors, whereas the high percentage of young donors reported in *Healthy People 2010* probably was due to a strong focus on recruitment among high school and college students [9].

Our results indicate a bimodal prevalence distribution with one peak at 25–30 years and a second smaller one at 50 years. We are not the first to show this distribution. A large study from England and Wales showed the same for both men and women [7]. Furthermore, an Icelandic study showed the same for men, but not for women [23]. For women, the bimodal distribution might be explained by a pause in donation in the years with pregnancies and small children. The male peak at around 50 years of age might be explained by earlier focus on recruitment at military facilities and male dominated workplaces or the fact that there is an increase in percentage of older donors [24].

Associations between blood donation and income and education have been observed in several studies [9; 12; 17; 16; 10]. It is a challenge for blood centers to recruit or maintain blood donors from socially less privileged groups. One study found that individuals with at least some college education donated at a prevalence of 8%, high school graduates donated at approximately 4%, and those who had not completed high school donated at only 2% [9]. These results are comparable to the Danish population (6.3%, 7.7%, and 3.6%, respectively). To our knowledge, the lower donor prevalence in the highest income group has not been described in previous studies. In Denmark there is a positive association between working hours and income. [25]

We hypothesize that the reason for this decrease in the highest income group, which was found both for men and women, might be related to the challenges of a busy working schedule. The highest income group had a lower prevalence of blood donation than the average donor, but blood donation is still dominated by individuals in the higher income groups.

Our data showed that the prevalence of blood donors was higher in urban than rural parts of the country, but differences were small. In Denmark, donation sites are concentrated in the larger cities and despite an effort to establish mobile donation sites across the country, the distance between donors and donation sites has increased [26]. Several studies report, that accessibility of the blood banks is important where inconvenient location prevented younger donors from returning [15; 16]. A study by Veldhuizen et al. showed that donors living in less urbanized areas, despite having to travel longer distances to donor facilities, were less likely to cease donation than donors from larger cities. The authors speculated that the likelihood of remaining a blood donor is influenced by factors other than having the opportunity to donate nearby [19].

In Denmark, 87.0% of the population has at least one Danish parent; for blood donors this proportion is 96.3%. In the Netherlands, 80.2% of the general population has at least one Dutch parent [12]. Data from *Healthy People 2010* showed that Caucasians donated at more than twice the prevalence of Asians, Hispanics, and African-Americans [9]. In Demark, there has been little focus on recruiting donors of non-Danish ethnicities compared to most other countries. This also means that the higher deferral rates among ethnic minorities compared to ethnically Danish individuals, as observed in other studies, is less critical in Denmark. Blood donors must be able to speak and read Danish to donate blood, which might prevent some groups from volunteering. With increasing internationalization of the Danish society, these rules might change. As mentioned earlier, Danish blood banks have stringent deferral policies, especially related to medicine use and travel.

The prevalence and relative risk of Danish blood donors varied considerably by cohabitation status, revealing distinct differences between men and women. For men, blood donation was strongly associated with living with a woman, whereas for women, cohabitation status only played a minor role in relation to blood donation. For men, there is a generally strong association between being married and having children on the one side and good health and high social status on the other. For women these associations are less clear. The association among men between blood donation and family life may reflect that good integration in society is a strong positive predictor of blood donation. According to Veldhuizen et al., blood donation is most common among married people [19].

The data presented here should be taken into account in blood donor recruitment plans. It is important to focus recruitment efforts on the groups most likely to respond positively, but it is also important to focus on larger groups, where recruitment campaigns can lead to a large number of new donors. One challenge is that the successful recruitment of young female donors is followed by losses of these same donors when they get children. Family friendly donation could be one way of overcoming this challenge. Another major challenge is the generally weak recruitment of socially marginalized groups as indicated by income, education, ethnicity and family status. Other ethnicities in Denmark are approximately 15% of the population (Table 1). Since this group is increasing both in Denmark and in most Western countries [7], we need to consider how to include them as blood donors, but a general change in recruitment strategy to motivate the socially marginalized groups might be challenging. Since no group has prevalence of blood donation above 10%, it might be useful focusing the recruitment on the more than 90% in the groups with both high prevalence and relative risk.

We recommend that the best investment in securing the future blood supply might be to focus on the very large group of donors with older children, since they constitute a very large proportion of the population and have a high prevalence of blood donation (Table 1). In addition, according to our results, they have a high prevalence of blood donation and both men and women at 40–50 years of age still have a lot of years as blood donors, and might have more spare time. [27]. When designing inclusion strategies, it is important to know the prevalence in the subgroups, but it is also important to know the average donation per donor in each subgroup. We found only very small differences in frequencies between the subgroups within each category in the Danish blood donor population.

Our observations are based on register-based information for an entire population, thus eliminating selection or measurement biases. Our study shows that the sociodemographic factors most strongly linked to blood donation are income levels among both women and men and cohabitation status among men. Having children and the age of the youngest child are associated with the prevalence of blood donation for both men and women. We recommend that this information is taken into account when planning donor recruitment and donor care and retention strategies not only in Denmark but also elsewhere as donor characteristics are likely to apply to other countries with comparable demographic and health care settings.
